# Intraoperative method of femoral head central measurement to prevent leg length discrepancy following hemiarthroplasty

**DOI:** 10.3389/fsurg.2022.1055199

**Published:** 2023-01-06

**Authors:** Hongxin Hu, Mei Lin, Xianwei Wu, Yujin Lin, Yijun Lin, Guoli Chen

**Affiliations:** ^1^ Department of Orthopaedics, Affiliated Hospital of Putian University, Putian, China; ^2^ The Third Clinical Medical College of Fujian Medical University, Putian, China; ^3^ Department of Surgery, Affiliated Hospital of Putian University, Putian, China; ^4^ Department of Radiology and Imaging, Affiliated Hospital of Putian University, Putian, China

**Keywords:** femoral neck fracture, hemiarthroplasty, femoral offset, intraoperative method of femoral head central measurement, leg length discrepancy

## Abstract

**Purpose:**

This study aimed to introduce and investigate the safety and efficiency of the intraoperative central measurement method of the femoral head (IM-CMFH) to prevent leg length discrepancies (LLD) after hemiarthroplasty.

**Methods:**

Overall, 79 patients aged 75 to 85 years with femoral neck fractures who underwent hemiarthroplasty were divided into two groups: the Control group (*n* = 46) and the IM-CMFH group (*n* = 33). The two groups were compared for postoperative LLD and the proportions of patients with greater than 10 mm, 6–10 mm, and within 5 mm, postoperative femoral offset (FO) difference and the proportions of patients within 5 mm, incremental greater than 5 mm and reduction greater than 5 mm. Next, the vertical distance from the center of the femoral head to the tip of the greater trochanter on the anatomical axis of the femur (VD-CFH-TGTAAF), leg length, and FO on the operative and non-operative sides within the IM-CMFH group. Finally, operative time, hemoglobin loss, Harris scores 3 months after surgery, and postoperative complications were analyzed.

**Results:**

Compared with the control group, the postoperative LLD and FO differences were significantly lower in the IM-CMFH group (*P *= 0.031; *P *= 0.012), and the proportion of patients with postoperative LLD greater than 10 mm decreased significantly (*P *= 0.041), while the proportion of patients with FO difference of within 5 mm increased (*P *= 0.009). In addition, there was no significant difference in the operative time, hemoglobin loss, and Harris score at 3 months postoperatively and postoperative complications between the two groups (*P *> 0.05). There was no significant difference in FO, leg-length, and VD-CFH-TGTAAF between the operative and non-operative sides within the IM-CMFH group (*P *> 0.05).

**Conclusion:**

Satisfactory results can be achieved by using the IM-CMFH to prevent LLD following hemiarthroplasty, and there is no increase in operative time, hemoglobin loss, or postoperative complications. This technique is efficient for hemiarthroplasties and is both simple and convenient.

## Introduction

Hemiarthroplasty (HA) is a common surgical procedure for treating femoral neck fractures in elderly patients. The incidence of femoral neck fractures in elderly patients is frequent, and treatments with HA procedures have significantly increased with the aging population (1, 2). Reconstruction of hip function through HA for femoral neck fractures may lead to pain relief, improved quality of life, and reduced rates of complications caused by the long-term effects of becoming bedridden after a fracture. A low rate of postoperative complications is the primary interest of all patients and is associated with a satisfactory outcome. The most concerning complications that are associated with either a total hip arthroplasty (THA) or HA are instability and leg length discrepancy (LLD) (3). Importantly, leg length is difficult to assess precisely during an operation (4, 5).

The occurrence of LLD is common, with an approximate incidence between 1%–27% after joint arthroplasty. The average LLD has been reported to vary between 3 mm and 17 mm (6). LLD may cause hip instability, ipsilateral knee pain, lower back pain, and prosthesis loosening. These effects can reduce patient satisfaction and even result in litigation (7, 8). Multiple intraoperative methods that are available to prevent LLD have been described; however, the entire patient population of these studies underwent a THA after being diagnosed with osteoarthritis, osteonecrosis, rheumatoid arthritis, or hip dysplasia (5, 7, 9–12). Femoral neck fractures were not observed or studied, and the LLD was measured preoperatively with fixed values. For femoral neck fractures, the preoperative LLD may change due to the displacement of the fracture. It is crucial to obtain a simple, convenient, and intraoperative technique to assess LLD during HA.

The current study introduces a new intraoperative method of central measurement of the femoral head (IM-CMFH) to prevent LLD following a HA procedure. The primary purpose of this study aims to (i) provide a novel approach to avoid and reduce the occurrence of LLDs, and (II) investigate its effificacy and safety in clinical application.

## Patients and methods

### Patients selection

Patients with femoral neck fractures who underwent a hemiarthroplasty from Jan 2018 to Jul 2020 at the Affiliated Hospital of our University were enrolled. Age, sex, preoperative complications, preoperative hemoglobin, and AO/OTA 31 types were collected.

**Figure 1:**
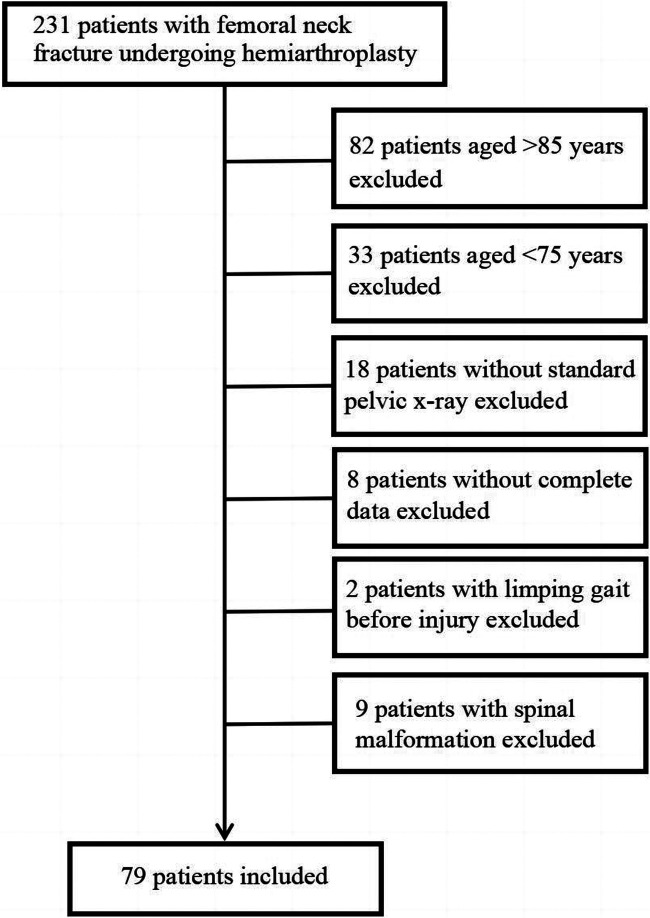
Flowchart demonstrating patient inclusion in the study.

This study followed the below-mentioned inclusion criteria: (1) diagnosed with femoral neck fractures; (2) patients between 75 and 85 years of age; and (3) patients who underwent unilateral hemiarthroplasty. However, the exclusion criteria consisted of (1) evidence of a pathological fracture, (2) incomplete data, (3) non-neutral pelvic x-ray, (4) limping gait before the injury, (5) spinal malformation (Figure 1). The study was carried out by the Declaration of Helsinki and its amendments. The Ethics Committee at our hospital approved the protocol (202152). Relevant guidelines and regulations are carried out for all methods. A written declaration of consent was obtained from all patients. Patients with the intraoperative method of central measurement of the femoral head were defined as IM-CMFH Group. Patients with the intraoperative method of the Shuck test, drop kick test, and the level of both knee joints was defined as the Control Group.

### Surgical technique

**Figure 2:**
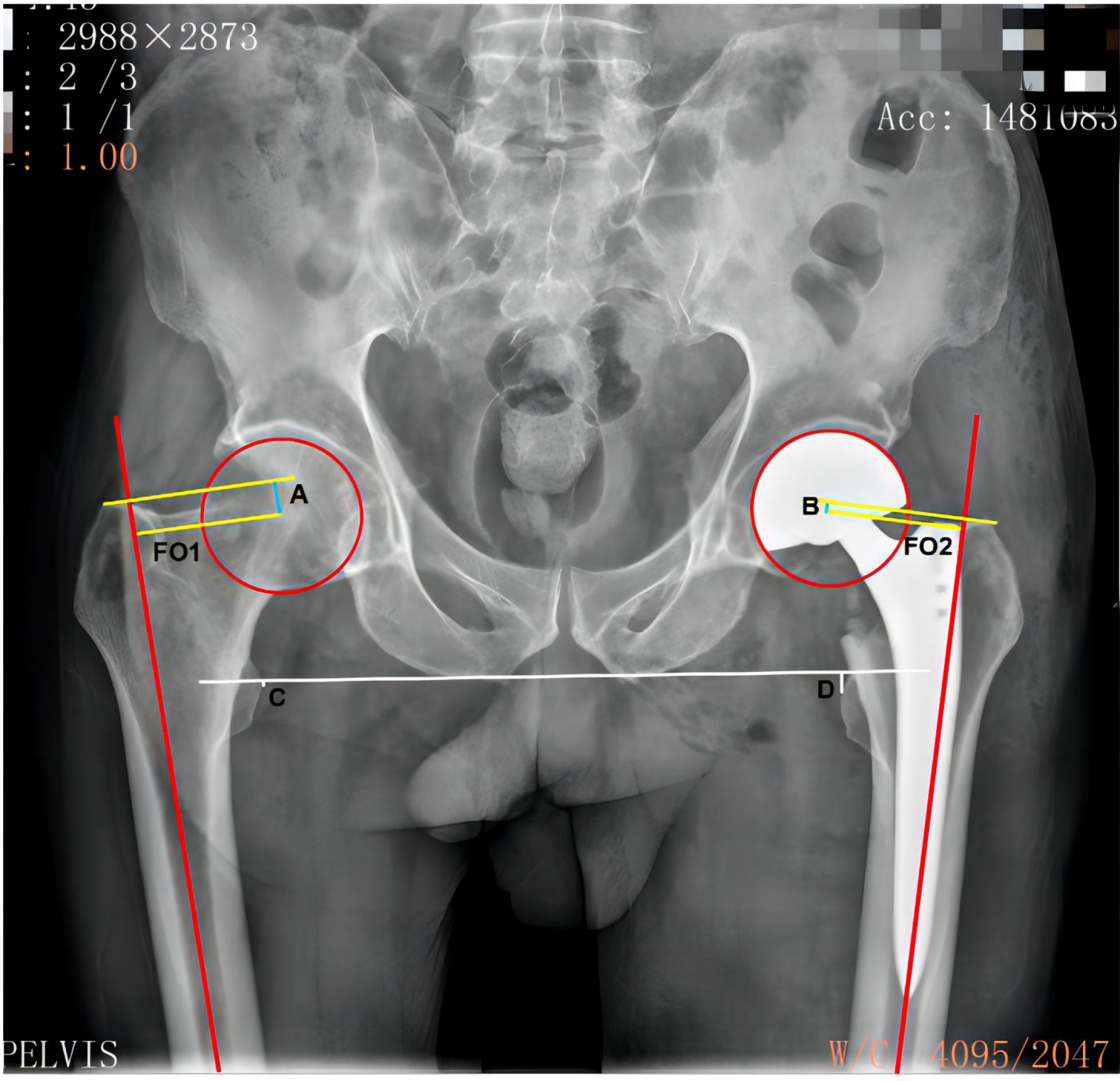
X-ray. (A) the vertical distance from the center of the femoral head to the tip of the greater trochanter on the anatomical axis of the femur on the non-operative side. (B) the vertical distance from the center of the femoral head to the tip of the greater trochanter on the anatomical axis of the femur after surgery on the operative side. (C), the vertical distance from the tip of the lesser trochanter to the connected line of the bilateral sciatic tuberosity after surgery on the non-operative side. (D) the vertical distance from the tip of the lesser trochanter to the connected line of bilateral sciatic tuberosity after surgery on the operative side. The LLD is the difference between the measured values of both sides. FO 1: the distance between the center of rotation of the femoral head and the long axis of the femur on the non-operative side. FO 2: the distance between the center of rotation of the femoral head and the long axis of the femur after surgery on the operative side. The difference between the measured values of the two sides is the FO difference. LLD, leg length discrepancy; FO, femoral offset.

**Figure 3:**
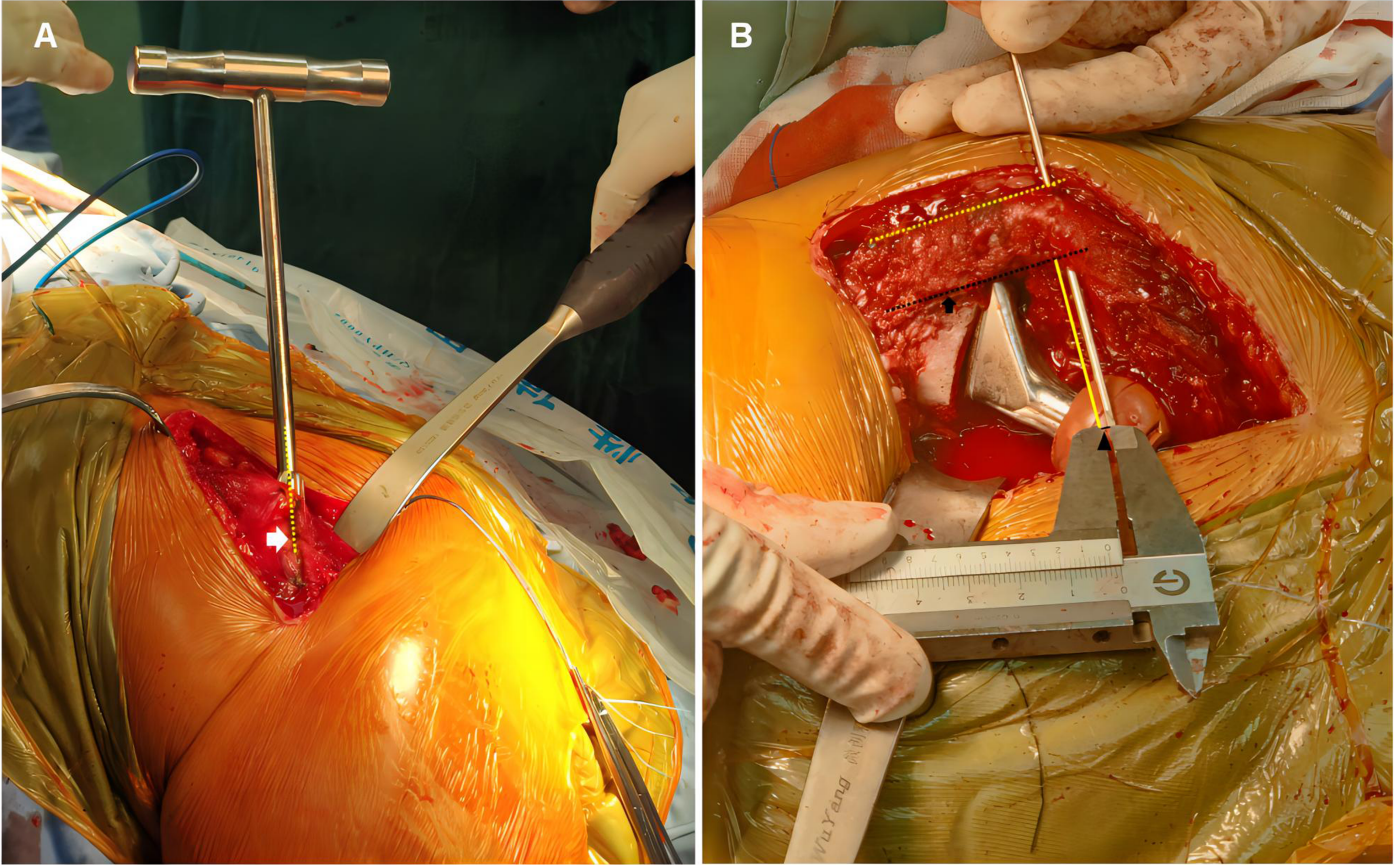
Intra-operative photograph. (**A**) Marking the line parallel to the reamer on the lateral of proximal femur for double checking (white arrow and yellow dotted line). (**B**) Measure the vertical distance from the center of the femoral head to the tip of the greater trochanter on the anatomical axis of the femur (black solid line and black arrowhead) and femoral offset (yellow solid line). Making the line parallel to the reamer on the lateral of proximal femur (yellow dotted line) and on the proximal femur representing long axis of femur (black arrow and black dotted line).

The anteroposterior pelvis radiographs were obtained preoperatively and postoperatively in a standardized fashion, with both hips extended and internally rotated 10 to 15 degrees (13), and the x-ray imaging magnification was calibrated to known coin size or the known bipolar shell size. The vertical distance from the center of the femoral head to the tip of the greater trochanter on the anatomical axis of the femur (VD-CFH-TGTAAF) and femoral offset (FO) was measured and recorded preoperatively on the nonoperative side (14) (Figure 2). All patients prepared for the surgery were administered combined spinal-epidural anesthesia and performed using the posterolateral approach in the lateral position by one of three senior orthopedic surgeons. The team observed a T-shaped joint capsule, and the femoral head was removed. The residual tissue and bone fragments were removed, and a suitable femoral stem trial mold was selected to enlarge the proximal femoral medullary cavity. After the matching trial components were inserted, the leg length was measured. The Control Group used the standard intraoperative techniques to assess leg length, including the Shuck test, drop kick test, and the level of both knee joints (15). IM-CMFH Group used intraoperative measurement of the VD-CFH-TGTAAF and FO (Figure 3). The method of IC-CMFH was as follows. The reamer was inserted into the medullary cavity of the femur, and two lines parallel to the reamer in the medullary cavity were marked on the proximal femur, one for double checking and one representing the long axis of the femur (Figures 3A,B). The medullary canal was reamed using tapered reamers of progressively increasing size. The femoral component was selected according to preoperative measurements. The highest point of the greater trochanter was found and the Kirschner wire was inserted against the apex of the bone surface of the greater trochanter, perpendicular to the marked parallel line (Figure 3B). The vertical distance from the center of the femoral head to the Kirschner wires and FO was measured (Figure 3B).

Appropriate measures were taken to adjust if there was a large difference between the two distances compared to the preoperative period. Measurements were adjusted as follows. (1) The VD-CFH-TGTAAF on the operative side was more than 2 mm shorter than on the non-operative side, indicating that the leg length was shorter than on the non-operative side. If the FO is shorter than the non-operative side, the FO and leg length could be increased by increasing the neck length (16). If the FO was longer than the non-operative side, the femoral stem with a large neck angle was chosen to increase the leg length and decrease the FO (16). (2) The VD-CFH-TGTAAF on the operative side is more than 2 mm longer than on the non-operative side, indicating that the leg length was longer than on the non-operative side. If the FO is shorter than on the non-operative side, the leg length may be reduced and the FO increased by further reaming and selecting a high offset femoral stem or by selecting a femoral stem with a small neck angle (16). If the FO was longer than on the non-operative side, a reduction in neck length was chosen to reduce the FO and leg length (16). (3) If the difference between the VD-CFH-TGTAAF on the surgical side and the non-surgical side was within 2 mm, which we consider acceptable, the prosthesis was adjusted according to the FO. Finally, a suitable femoral stem prosthesis and femoral head were placed.

### Methods of assessment

Routine blood tests were performed on the first-day post-surgery to determine the patient's hemoglobin levels. Conventional low molecular weight heparin anticoagulation was administered 12 h after the surgery and continued until discharge to prevent deep vein thrombosis. Patients were started with an ankle-pump, quadriceps-contraction exercises, and weight-bearing walking with the aid of a walker on the 1st-day post-surgery. An x-ray examination was performed in the first week, and the VD-CFH-TGTAAF and FO were measured and recorded (Figure 2).

The leg length was obtained as the distance between the lines that connect the lowest edge of the right and left ischial tuberosity to the most prominent point of the lesser trochanter (17, 18). The FO was defined as the vertical distance from the center offemoral head to the long axis of femur on x-ray (14, 19, 20). Leg length, VD-CFH-TGTAAF, and FO of the operative side and nonoperative side were measured using a computerized imaging system by the radiologist without knowing the grouping. Three measurements of each value were averaged, and the results of each measurement were reconfirmed by two senior orthopedic surgeons. The LLD is the absolute value of the difference in leg length between the operative side and the nonoperative side, and the FO difference is the absolute value of the difference in FO between the operative side compared to the nonoperative side. According to the grouping of Sato et al. (14), we divided the FO difference into three groups: within 5 mm of the absolute value on the operated side compared to the contralateral side, reduced by >5 mm on the operated side compared to the contralateral side, and increased by >5 mm on the operated side compared to the contralateral side. The study's lead researcher recorded all measurements in millimeters using a computerized imaging system by the radiologist.

The operative time (from the skin incision to the wound closure), the hemoglobin loss (hemoglobin on the first postoperative day subtract preoperative hemoglobin), and postoperative complications were recorded. Harris scores at 3 months after surgery was calculated to evaluate therapeutic efficacy.

### Statistical analysis

The *χ*^2^, *F*-test, and Mann–Whitney *U*-test were used to compare the differences between the two groups in demographic characteristics. Count data were expressed as numbers (percentage), and differences between two groups were compared by chi-square test. Differences in continuous variables between groups were analyzed using the Student's t-test if they obeyed a normal distribution and expressed as mean ± standard deviation (SD); otherwise, the Mann–Whitney *U*-test was used and expressed as medians [interquartile ranges (IQR)]. All analyses were performed using SPSS version 22.0, *P *< 0.05 was considered statistically significant. Power calculation was analyzed using the PASS (version 15.0.5). In the present study, 33 cases and 46 controls can achieve a statistical power of 0.81785 to calculate the LLD and 0.89756 to calculate the FO.

## Results

### Demographic characteristics

Seventy-nine cases of femoral neck fractures were enrolled. According to the intraoperative method of assessing the leg length, 46 cases were included in the control group, and 33 cases were included in the IM-CFH group. There was no statistical difference in age, sex, preoperative complications, and AO/OTA 31 types between the two groups (Table 1).

**Table 1:** Demographic characteristics of all patients.

Variable	Control group (*n* = 46)	IM-CMFH Group (*n* = 33)	Statistic value	*P* value
Sex (male/female)	18/28	8/25	1.929*	0.165
Age (years)	81.00 (78.00, 83.00)	81.00 (78.00, 82.50)	−0.060^&^	0.952
Hypertension	18	20	3.550*	0.060
Diabetes	9	7	0.032*	0.857
Preoperative hemoglobin (g/L)	120.04 ± 14.38	116.18 ± 18.29	−1.050^#^	0.297
AO/OTA type 31-B1	13	4	2.964*	0.085
AO/OTA type 31-B2	17	17	1.661*	0.197
AO/OTA type 31-B3	16	12	0.021*	0.885

IM-CMFH: intraoperative method of central measurement of the femoral head.

* *χ*^2^ test.

^&^ Mann–Whitney *U*-test and expressed as median (IQR).

^#^ Independent-samples *t*-test and expressed as mean ± SD.

### The LLD and FO

The median number of postoperative LLD was 4.70 mm (IQR, 2.77–9.45 mm) in the control group and 3.30 mm (IQR, 1.50–5.40 mm) in the IM-CFH group. The postoperative LLD in IM-CFH group was significantly less compared to the control group (*P *= 0.031) (Table 2). The LLD was classified into three groups: within 5 mm, 5–10 mm, and over 10 mm (15). In the postoperative LLD within 5 mm, there were 24 cases in the control group and 23 cases in the IM-CFH group; between 5 and 10 mm, there were 12 cases in the control group and 9 cases in the IM-CFH group, and there was no statistical difference between the two groups (*P *> 0.05). While on LLD over 10 mm, a larger proportion of patients in the control group (21.74%, 10/46) compared to only 3.03% (1/33) in the IM-CFH group (*P *= 0.041) (Table 2).

**Table 2:** Postoperative LLD and FO of radiographic.

Variable	Control Group (*n* = 46)	IM-CMFH Group (*n* = 33)	Statistic value	*P* value
LLD (mm)	4.70 (2.77, 9.45)	3.30 (1.50, 5.40)	−2.157^&^	0.031
LLD <5 mm (number, rate)	24 (52.17%)	23 (69.70%)	2.448*	0.118
LLD 5–10 mm (number, rate)	12 (26.09%)	9 (27.27%)	0.093*	0.760
LLD >10 mm (number, rate)	10 (21.74%)	1 (3.03%)	4.159^#^	0.041
FO difference (mm)	5.20 (2.48, 8.60)	3.00 (1.00, 4.95)	-2.510^&^	0.012
FO difference within 5 mm (number, rate)	23 (50.00%)	26 (78.79%)	6.761*	0.009
FO difference more than 5 mm reduction (number, rate)	7 (15.22%)	3 (9.09%)	0.216^#^	0.642
FO difference more than 5 mm increment (number, rate)	16 (34.78%)	4 (12.12%)	5.219*	0.022

LLD, leg length discrepancy; FO, femoral offset; IM-CMFH, intraoperative method of central measurement of the femoral head. The LLD is the absolute value of the difference in leg length between the operative side and the nonoperative side, and the FO difference is the absolute value of the difference in FO between the operative side compared to the nonoperative side. The FO difference within 5 mm was defined as the absolute value of the difference between the FO on the operative side and the non-operative side was within 5 mm. FO difference more than 5 mm reduction was defined as the FO on the nonoperative side minus FO on the operative side is more than 5 mm. FO difference more than 5 mm increment was defined as the FO on the operative side minus FO on the nonoperative side is more than 5 mm.

* *χ*^2^ test.

^&^ Mann–Whitney *U*-test and expressed as median (IQR).

^#^ Continuity correction of *χ*^2^ test.

In addition, we also compared the postoperative FO difference between the two groups. The median number of postoperative FO difference was 5.20 mm (IQR, 2.48–8.60 mm) in the control group and 3.00 mm (IQR, 1.00–4.95 mm) in the IM-CFH group, with a significant difference between the two groups (*P *= 0.012) (Table 2). There were 23 cases in the control group and 26 in the IM-CFH group within 5 mm of the FO difference, which would have a statistically significant difference between the two groups (*P *= 0.009) (Table 2). More than 5 mm reduction, there were 7 cases in the control group and 3 cases in the IM-CFH group, and there was no statistically significant difference between the two groups (*P *= 0.642) (Table 2). With more than 5 mm increment, there was a larger proportion of patients in the control group (34.78%, 16/46), but only 12.12% (4/33) in the IM-CFH group (*P *= 0.022) (Table 2).

Next, we compared the leg length, FO, and VD-CFH-TGTAAF between the operative and nonoperative sides of the IM-CMFH group (Table 3). As shown in Table 3, the median number of VD-CFH-TGTAAF, leg length, and FO on the nonoperative side were 6.35 mm (IQR, 4.33–9.75 mm), 15.10 mm (IQR, 12.30–20.11 mm), and 49.20 mm (IQR, 46.50–52.70 mm), respectively, and the operative side was 5.50 mm (IQR, 4.22–6.82 mm), 13.10 mm (IQR, 9.30–18.07 mm), and 51.60 mm (IQR, 47.50–54.85 mm), and the differences between the operative and nonoperative sides did not reach statistical significance (*P *= 0.182; *P *= 0.079; *P *= 0.278).

**Table 3:** Comparison of VD-CFH-TGTAAF, leg length, and FO of the operative side and the nonoperative side in IM-CMFH group.

Variable	Nonoperative Side	Operative Side	Statistic value	*P* value
VD-CFH-TGTAAF (mm)	6.35(4.33, 9.75)	5.50(4.22, 6.82)	−1.334*	0.182
Leg length (mm)	15.10 (12.30, 20.11)	13.10 (9.30, 18.07)	−1.757*	0.079
FO (mm)	49.20(46.50, 52.70)	51.60(47.50, 54.85)	−1.084*	0.278

IM-CMFH, intraoperative method of central measurement of the femoral head; VD-CFH-TGTAAF, vertical distance from the center of the femoral head to the tip of the greater trochanter on the anatomical axis of the femur; FO, femoral offset.

* Mann–Whitney *U*-test and expressed as median (IQR).

### Operative time, the hemoglobin loss, complication, and Harris score

Finally, we compared the operative time, hemoglobin loss, postoperative complications, and Harris scores at 3 months postoperatively between the two groups (Table 4). In the control group, the median number of operative time was 85.00 min (IQR, 70.00–100.00 min), the hemoglobin loss was 19.00 g/L (IQR, 11.00–24.25 g/L), and the Harris score at 3 months postoperatively was 80.00 (IQR, 77.00–83.00). While in the IM-CMFH group, the median number of operative time was 80.00 min (IQR, 70.00–90.00 min), the hemoglobin loss was 19.00 g/L (IQR, 8.00–22.00 g/L), and the Harris score at 3 months postoperatively was 80.00 (IQR, 77.00–83.00), with no significant differences between the two groups (Table 4). The postoperative complication rate in the control group was 6.52% (3/46): 2 cases of intermuscular vein embolism and 1 case of dislocation, successfully repositioned by manipulation. The postoperative complication rate in the IM-CMFH group was 3.03% (1/33): 1 case of intermuscular vein embolism. There was no significant difference in the incidence of postoperative complications between the two groups.

**Table 4:** Comparison of the operative time, hemoglobin loss, postoperative complications, and harris score.

Variable	Control Group	IM-CMFH Group	Statistic value	*P* value
Operative time (min)	85.00 (70.00, 100.00)	80.00 (70.00, 90.00)	−1.652^&^	0.099
Hemoglobin loss (g/L)	18.47 ± 9.00	17.30 ± 10.29	0.537^#^	0.592
Postoperative complications	3 (6.52%)	1 (3.03%)	0.333*	0.564
Harris score	80.00 (77.00, 83.00)	80.00 (77.00, 83.00)	−0.495^&^	0.621

IM-CMFH, intraoperative method of central measurement of the femoral head.

* Continuity Correction of *χ*^2^ test.

^&^ Mann–Whitney *U*-test and expressed as median (IQR).

^#^ Independent-samples *t*-test and expressed as mean ± SD.

## Discussion

Leg length equality is an important factor correlated with healthy joint arthroplasty. In the current study, the intraoperative VD-CFH-TGTAAF and FO were compared with that of the nonoperative side before the surgery. The results showed that compared with the control group, the IM-CMFH group had a reduced proportion of LLD, especially for LLD greater than 10 mm, and a smaller FO difference, without increasing operative time, hemoglobin loss, or postoperative complications.

The incidence of LLD is high, is inconsistent in different types of literature, and can cause some complications (6–8). LLD must be 10 mm or less in a patient for optimal recovery and quality of life (8). Despite careful attention, unexpected discrepancies up to 10 mm occasionally occur. Possible causes are sinking of the stalk, preoperative hip flexion contracture, accurate preoperative planning based on x-rays at different magnifications, and inexperienced surgeons. Small differences can lead to dissatisfaction in a few patients (7). Significant leg length inequality after arthroplasty is a major cause of patient dissatisfaction due to abnormal gait mechanics resulting in knee and back pain, early prosthesis loosening, and revision surgery (21). The patients with LLD greater than 10 mm had significantly worse Oxford hip scores (22), and postoperative LLD or offset differences greater than 5 mm were associated with nonphysiologically gait kinematics (23).

Currently, there is no standard method to assess the length of the lower limbs, and it is critical to find a method to prevent LLD. Some intraoperative techniques have been proposed to evaluate leg length. However, contrasting results have been presented in the literature. For example, previously published techniques include the patellae and tibiae (5), Steinmann pin combined with suture (10), the principal component analysis (PCA) leg lengthening gauge (7), the use of an “L” shaped caliper (11), and intraoperative fluoroscopy (24). The previous research participants in studies concerning LLD included those suffering from osteoarthritis, osteonecrosis, rheumatoid arthritis, or hip dysplasia. In the current study, the patients had experienced femoral neck fractures with displacement, causing the preoperative LLD. In patients with femoral neck fractures, it is difficult to assess how much elongation is required to achieve equilibrium between the lower extremities. Our study applied a method explicitly designed to focus on the VD-CFH-TGTAAF and FO on the non-operative side, regardless of the preoperative LLD. Intraoperatively, we measured the VD-CFH-TGTAAF and FO on the operative side, compared them with the non-operative side measured preoperatively, and made adjustments.

In the current study, the IM-CMFH method was used to assess intraoperative leg length, and the postoperative LLD (3.91 mm) was similar to 3.64 mm obtained by the intraoperative use of calipers by Fansur et al. (25), and only one case had a postoperative LLD greater than 10 mm, which was significantly lower than that of the control group, with a ratio similar to that reported by Dasai et al. (15). The difference was not statistically significant by comparing the leg length of the own operative side and the non-operative side.

The inadequate offset may be associated with poor gait patterns, poor functional outcomes, impingement, pain, increased muscle strength and fatigue, increased joint reaction forces, and dislocation (26–29). The excessive offset was related to increased pain, wear and tear, and significant leg length inequality (30). It has been found that FO reduction greater than 5 mm is associated with hip abductor muscle weakness (14). In this study, compared with the control group, the FO difference was significantly reduced in the IM-CMFH group, and the proportion of FO differences within 5 mm was relatively high. The postoperative FO in the group using the IM-CMFH method was similar to the 3.96 mm obtained intraoperatively using A mechanical measurement device by Barbier et al. (31). In contrast, the proportion of more than 5 mm reduction and increment was low. We found that the FO was similar by comparing the operative and non-operative sides of IM-CMFH group, and the difference was not statistically significant.

Although there were differences in postoperative LLD and FO difference between the two groups, there was no difference in Harris score at 3 months postoperatively, which may be related to the use of walking aids by patients in the early postoperative period. Whitehouse et al. (32) noted no significant correlation between postoperative structural LLD and functional outcome, while abductor weakness due to FO generally occurs at 6–12 months and does not change early (33). Therefore, there was no significant difference in Harris score at 3 months postoperatively in our study. A longer follow-up is needed to validate further the effects of LLD and FO on clinical function and gait.

However, there are some limitations to this research. Firstly, this is a retrospective case study subjected to inherent limitations. Secondly, radiographic measurements are dependent on position and magnification. The measurement of FO and leg length is influenced by pelvic tilt and femoral rotation (12, 34). Although the accuracy of radiological measurements may be poor compared to computed tomodensitometry (CT) (18). To minimize bias, all radiographs were taken in the same radiology department with the same standardized technique, ensuring 10–15° of internal rotation of both femurs. Thirdly, the function and pain in the early postoperative period showed no correlation with postoperative LLD and FO (32, 33, 35), and a longer follow-up is required in order to assess the impact of FO and LLD on function. Finally, our technique still requires a learning curve to be able to do the various intraoperative markings properly.

## Conclusions

In summary, this study has identified a technique for measuring the VD-CFH-TGTAAF intraoperative to avoid LLD. It is a simple, easy-to-adopt, and convenient assessment of LLD. By adopting the new technology, we have shown that we can accurately and efficiently determine the leg length. And there is no increase in operative time, hemoglobin loss, or postoperative complications.

## Data Availability

The raw data supporting the conclusions of this article will be made available by the authors, without undue reservation.
